# DELP-Net: A Differentiable Entropy Layer Pyramid Network for End-to-End Low-Rate DoS Detection

**DOI:** 10.3390/e28030328

**Published:** 2026-03-15

**Authors:** Jinyi Wang, Congyuan Xu, Jun Yang

**Affiliations:** 1 College of Artificial Intelligence, Jiaxing University, Jiaxing 314001, China; 00199685@stu.zjxu.edu.cn (J.W.); juneryoung@zjxu.edu.cn (J.Y.); 2 College of Computer Science and Technology, Zhejiang University, Hangzhou 310058, China

**Keywords:** intrusion detection, cybersecurity uncertainty, Rényi entropy, pyramid fusion, deep learning

## Abstract

Low-rate Denial-of-Service (LDoS) attacks exploit periodic traffic pulses to trigger congestion while maintaining a low average rate, making them highly stealthy and difficult to distinguish from legitimate bursty traffic using threshold-based or simple statistical detectors. To address this challenge, this paper proposes DELP-Net, an end-to-end Differentiable Entropy Layer Pyramid Network for window-level online LDoS detection directly from raw traffic. DELP-Net combines a multi-scale one-dimensional convolutional pyramid with a differentiable Rényi-entropy-driven attention mechanism to capture distributional regularity and weak repetitive patterns characteristic of LDoS traffic. In addition, an entropy-conditioned temporal convolutional network is employed to model cross-window periodic dependencies in a lightweight manner, together with an entropy-regularized hybrid loss to enhance robustness under complex background traffic. Experiments on the low-rate DoS dataset show that DELP-Net achieves an average F1 score of 0.9877 across six LDoS attack types, with a detection rate of 98.69% and a false-positive rate of 1.15%, demonstrating its effectiveness and suitability for practical online intrusion detection deployments.

## 1. Introduction

Low-rate Denial-of-Service (LDoS) attacks represent a class of highly stealthy yet destructive network attacks. Unlike conventional flooding-based Distributed Denial-of-Service (DDoS) attacks, LDoS attacks typically inject periodic traffic pulses at carefully selected moments to trigger congestion or resource bottlenecks [1,2]. By exploiting protocol dynamics and queue behaviors, these attacks can continuously suppress the throughput of legitimate services and even cause service unavailability while maintaining a very low average traffic rate. As a result, detection mechanisms that rely on traffic amplitude thresholds or simple statistical indicators often fail to respond effectively. Moreover, real-world network traffic exhibits inherent burstiness and diversity [3]. Legitimate phenomena such as flash crowds, application-level request bursts, link jitter, and retransmissions further increase detection difficulty, causing traditional statistical detectors to suffer from high false-positive rates and poor generalization.

Information entropy provides a powerful theoretical tool for network security analysis. Entropy characterizes the uncertainty and distributional complexity of random variables and has been widely used to quantify variations in source addresses, ports, packet lengths, inter-arrival times, and protocol field patterns [4]. In the context of LDoS attacks, traffic during pulse phases typically exhibits stronger regularity and repetitiveness [5]. Such aggregation effects induce observable changes in entropy-related measures. Compared with Shannon entropy [6], Rényi entropy [7] introduces a tunable order parameter α to adjust sensitivity to different regions of a distribution. When α>1, Rényi entropy becomes more sensitive to high-probability events, which makes it particularly suitable for amplifying distributional concentration caused by repetitive structures. This property aligns well with the statistical characteristics of LDoS traffic under low-rate conditions.

In recent years, deep learning has been extensively applied to intrusion detection and traffic classification [8], with representative models including convolutional neural networks (CNNs) [9], recurrent neural networks (RNNs) [10], temporal convolutional networks (TCNs) [11], and attention-based architectures [12]. One line of research relies on tools such as CICFlowMeter to extract flow-level statistical features before training classifiers [13]. Although these approaches achieve good performance on benchmark datasets, they suffer from feature engineering bottlenecks and complex deployment pipelines. Another line of work attempts end-to-end modeling from raw bytes or packet sequences. However, many existing methods treat network traffic as generic sequential signals and commonly employ amplitude- or mean-driven attention mechanisms, such as Squeeze-and-Excitation (SE) [14] or Convolutional Block Attention Module (CBAM) [15]. These attention mechanisms tend to emphasize channels with high energy responses, whereas the key cues of LDoS attacks are often not high-magnitude signals, but rather subtle distributional regularity and entropy anomalies. Consequently, how to elevate entropy from an external statistical descriptor to an internal, learnable operator within deep networks, while maintaining a lightweight and deployment-friendly design, remains a challenging and insufficiently explored problem.

To address these challenges, this paper proposes DELP-Net, an end-to-end differentiable entropy pyramid network for LDoS detection. The proposed network adopts time-window slicing as the basic data organization strategy, transforming raw network traffic into fixed-shape window tensors that are directly fed into a deep model to produce window-level predictions. DELP-Net employs a multi-scale one-dimensional convolutional pyramid to extract representations with diverse receptive fields, covering byte-level local patterns, protocol field structures, and intra-window packet sequence characteristics. On top of this, we design a Differentiable Rényi Entropy Attention Module (DREAM), which embeds differentiable Rényi entropy computation into intermediate network layers and performs entropy-driven channel recalibration. This design enables the model to adaptively focus on entropy-abnormal channels and informative segments. To further capture periodic pulse dependencies across windows, an entropy-conditioned TCN is introduced for temporal modeling. Dilated convolutions are used to obtain large receptive fields with low inference overhead. Finally, an entropy-regularized hybrid loss is incorporated to enhance the separability between attack and benign samples in the entropy domain, thereby improving robustness and reducing false positives under complex background traffic.

The main contributions of this paper are summarized as follows.

1. We propose DELP-Net, an end-to-end differentiable entropy pyramid network for low-rate Denial-of-Service (LDoS) detection. The proposed approach operates directly on raw traffic streams segmented into time windows and upgrades information entropy from an external statistical descriptor to an intrinsic, learnable operator within the network, establishing a unified information-theoretic deep learning framework for detecting low-rate and weak-signal attacks.
2. We design a Differentiable Rényi Entropy Attention Module (DREAM), which embeds Rényi entropy computation into the forward and backward propagation of the network and leverages entropy-driven channel recalibration. By characterizing distributional concentration and repetitive patterns, DREAM enhances the model’s sensitivity to weak and regular LDoS behaviors, outperforming conventional amplitude- or mean-based attention mechanisms.
3. We introduce a multi-scale entropy pyramid learning framework that jointly models feature representations and entropy statistics across multiple receptive-field scales. Through scale-wise entropy-aware fusion, the proposed framework effectively captures both short burst behaviors and long-period structures of LDoS attacks, improving robustness to diverse low-rate attack patterns.
4. We develop an entropy-conditioned temporal convolutional network (TCN) for cross-window temporal modeling, in which entropy-guided gating mechanisms are used to modulate dilated convolutional responses. This design enables the model to focus on entropy anomalies and abrupt entropy variations over time, facilitating effective modeling of periodic pulses and long-range dependencies in LDoS traffic.

The remainder of this paper is organized as follows. Section 2 reviews related work. Section 3 presents the overall methodology of DELP-Net. Section 4 describes the experimental setup and main results. Section 5 further validates the effectiveness of the proposed method through ablation studies, parameter sensitivity analysis, robustness analysis, and feature visualization. Finally, Section 6 concludes the paper.

## 2. Related Work

LDoS attacks are characterized by low average traffic rates, strong periodicity, and high similarity to legitimate bursty traffic. These properties distinguish LDoS detection from traditional flooding-based DoS detection in terms of modeling objectives, feature selection, and deployment constraints [16]. Existing studies on LDoS detection can be broadly categorized into three groups: statistical and signal processing-based methods, traditional machine learning-based methods, and deep learning-based methods.

### 2.1. Statistical and Signal Processing-Based LDoS Detection

Early studies on LDoS detection primarily adopt perspectives from signal processing and statistical analysis, treating network traffic as time series or stochastic processes and exploiting periodic pulse characteristics for detection. Representative approaches include power spectral density analysis in the frequency domain [17], short-time Fourier transform [18], and wavelet transform [19]. These methods typically identify anomalies by detecting low-frequency energy aggregation or periodic peaks, and are effective to some extent in scenarios where attack periodicity remains stable.

In parallel, information entropy has been applied to anomaly detection tasks by measuring uncertainty variations in attribute distributions such as source addresses, ports, packet lengths, and inter-arrival times. Considering the low-rate and high-regularity nature of LDoS attacks, some studies further introduce generalized entropy measures, including Rényi entropy and Tsallis entropy, to enhance sensitivity to distributional concentration and repetitive structures [20,21,22].

Despite their low computational cost and ease of implementation, statistical and signal processing-based methods face notable limitations in real-world network environments. First, these approaches often rely on manually designed features and fixed thresholds. Background traffic burstiness and jitter can significantly perturb statistical measures, making false-positive rates difficult to control. Second, periodic characteristics may be intentionally weakened through attack parameter randomization, such as varying pulse periods, duty cycles, or injecting jitter, which degrades the reliability of frequency-domain and threshold-based detectors. Third, statistical methods usually operate on low-dimensional attribute distributions and fail to exploit fine-grained structural information in protocol fields and payload fragments, resulting in limited expressive power against complex attack variants. These limitations indicate that relying solely on external statistical indicators is insufficient for robust LDoS detection under complex traffic conditions.

### 2.2. Traditional Machine Learning for LDoS and DoS Detection

To overcome the rigidity of fixed thresholds and single statistical metrics, traditional machine learning-based approaches typically adopt a two-stage pipeline [23]. In the first stage, handcrafted statistical features are extracted from PCAP files or flow records. In the second stage, classifiers are trained to perform detection. Common feature extraction tools generate flow-level or window-level feature vectors that include packet counts, byte counts, flow durations, inter-arrival time statistics, directional statistics, flag counts, and packet length quantiles [24,25,26].

In terms of classifiers, support vector machines [27], random forests [28], gradient boosting trees [29], autoencoders [30] and ensemble learning [31] methods have achieved relatively high accuracy on intrusion detection benchmark datasets. These models also provide a degree of nonlinear discrimination capability and feature importance analysis.

However, the major bottleneck of this category lies in feature engineering and deployment complexity. First, handcrafted features heavily depend on expert knowledge and often fail to cover diverse LDoS implementations. Attackers can adjust pulse patterns or perturb protocol fields to make statistical features resemble benign traffic, leading to degraded detection performance. Second, feature extraction usually requires complex preprocessing pipelines, introducing additional latency and maintenance overhead, and posing consistency risks between offline training and online deployment. Third, fixed feature spaces constrain model adaptability, causing generalization performance to deteriorate when network environments, application behaviors, or sampling conditions change. These issues limit the applicability of traditional machine learning frameworks for end-to-end, low-latency, and robust online LDoS detection.

### 2.3. Deep Learning Based Intrusion Detection

Deep learning provides end-to-end representation learning capabilities for intrusion detection. In existing research, CNNs are commonly used to capture local patterns with efficient inference, RNNs are employed to model temporal dependencies, and TCNs leverage dilated convolutions to achieve large receptive fields while preserving parallel computation [32,33,34]. Transformer and self-attention architectures are further introduced to learn global dependencies [35,36,37].

Regarding data representation, many studies still rely on handcrafted statistical features as model inputs, while others attempt to map traffic into image-like representations or directly model raw byte sequences in an end-to-end manner. To enhance discriminative ability, attention mechanisms such as SE and CBAM are frequently incorporated for channel recalibration, improving feature selection and information aggregation [38].

Although deep models achieve notable performance gains on several benchmark datasets, structural limitations remain for LDoS detection. First, many approaches still depend on engineered feature inputs, and end-to-end learning directly from raw network traffic remains relatively underexplored, limiting the utilization of fine-grained protocol fingerprints and payload structures. Second, mainstream attention modules usually rely on mean, amplitude, or correlation statistics, which tend to emphasize high-energy responses. In contrast, the critical cues of LDoS attacks often manifest as distributional regularity and entropy anomalies rather than prominent amplitude changes. Third, some methods adopt heavy sequence modeling backbones, leading to high inference overhead that is incompatible with latency and resource constraints in high-throughput online environments.

These observations suggest the need for an end-to-end framework that integrates information entropy as an internal, learnable operator and cooperates with lightweight temporal modeling, enabling stable perception of weak LDoS signals under complex background traffic while remaining suitable for practical deployment.

### 2.4. Recent Deep Learning Advances in IDS

In recent years, more advanced deep learning architectures have been explored for network intrusion detection. Transformer-based models have attracted increasing attention due to their ability to capture long-range dependencies and complex feature interactions in network traffic. By leveraging self-attention mechanisms, these models can automatically learn contextual relationships among traffic features and improve detection accuracy for sophisticated attacks. For example, Long et al. [39] proposed a transformer-based intrusion detection approach for cloud environments, demonstrating the effectiveness of attention mechanisms in modeling network traffic patterns. Xi et al. [40] further introduced a multi-scale transformer architecture to capture traffic features at different temporal scales, which improves detection performance on complex intrusion patterns. Recent studies have also explored self-supervised transformer-based representation learning to enhance generalization capability when labeled traffic data is limited [41]. These studies highlight the strong representation ability of transformer architectures for network security tasks. However, transformer-based IDS models often involve higher computational cost and require larger training datasets.

Another emerging research direction focuses on graph-based cybersecurity models. Since network communications naturally form relational structures among hosts, flows, and services, several studies model network traffic as graphs and apply graph neural networks to learn structural patterns of attacks. For instance, Lo et al. [42] proposed E-GraphSAGE, a graph neural network-based intrusion detection method that captures both flow features and network topology. Sun et al. [43] presented a graph neural network intrusion detection framework that integrates dynamic runtime telemetry with attack graph information. Graph-based approaches are also widely used for modeling communication relationships and multi-stage attack behaviors in complex networks [44]. Although these models can effectively capture relational dependencies among network entities, they typically require explicit graph construction and introduce additional preprocessing overhead.

Compared with these approaches, the method proposed in this work focuses on lightweight traffic representation and efficient feature extraction from flow-based traffic samples. The proposed architecture combines entropy-aware feature modeling with temporal convolutional learning to capture subtle traffic dynamics associated with low-rate DoS behaviors while maintaining relatively low computational complexity for practical deployment.

## 3. Methodology

### 3.1. Input Representation

This study focuses on the end-to-end detection of low-rate Denial-of-Service attacks. Given raw network traffic data, the input can be obtained from PCAP files captured at mirror ports or from equivalent online traffic streams. The model takes time-window-sliced raw data as input and directly outputs window-level attack labels, thereby realizing an end-to-end mapping from raw traffic to labels.

Let the time-ordered packet sequence obtained from packet capture be denoted as $P={\left\{p_{i}\right\}}_{i=1}^{N_{p}}$, where each packet $p_{i}$ is associated with a timestamp $t_{i}$ and a byte sequence $b_{i}\in {\left\{0,1,\ldots ,255\right\}}^{\ell_{i}}$. A fixed-length sliding time window is applied to segment P. Let the window length be $T_{w}$ and the stride be $T_{s}$. The *n*-th time window is defined as $W_{n}=\left[\left(n-1\right)T_{s},\left(n-1\right)T_{s}+T_{w}\right)$. The set of packets within this window is given by

(1) $$P_{n}=\left\{p_{i}\in P|t_{i}\in W_{n}\right\}.$$

To satisfy the fixed-size input requirement of neural networks, at most *K* packets are selected within each time window, and each packet is truncated or zero-padded to a fixed byte length *L*. Let TrimPad(·,L) denote the truncation and padding operator. The input tensor corresponding to window $W_{n}$ is constructed as $X_{n}\in R^{K\times L}$:

(2) $$X_{n}\left[k,:\right]=\frac{1}{255}\cdot \mathrm{TrimPad}\left(b_{\left(n,k\right)},L\right),\;k=1,2,\ldots ,K,$$

where $b_{\left(n,k\right)}$ denotes the byte sequence of the *k*-th packet selected from $P_{n}$ in chronological order. If the number of packets in the window is smaller than *K*, the remaining rows are padded with zeros. This representation preserves protocol header fields and partial payload information at the byte level while enabling low-latency streaming construction in practical deployments.

Window-level labels are used as supervision. Let $T_{a}\left(W_{n}\right)$ denote the duration of attack traffic overlapping with window $W_{n}$, and define the attack ratio as $r_{n}=T_{a}\left(W_{n}\right)/T_{w}$. The window label $y_{n}\in \left\{0,1\right\}$ is determined by

(3) $$y_{n}=\left\{\begin{array}{c} 1,\;r_{n}\geq \tau \\ 0,\;r_{n}<\tau \end{array}\right.,$$

where τ is a predefined label-alignment threshold. For offline experiments on public datasets, $T_{a}\left(W_{n}\right)$ can be computed from original annotations or attack start and end times. For online environments, $y_{n}$ can be generated from security alerts or injected attack scripts. With the sliding-window formulation, the model produces a prediction every stride $T_{s}$, supporting continuous detection and early warning.

Algorithm 1 summarizes the complete procedure of sliding-window segmentation, fixed-shape tensor construction, and window-level label alignment.

**Algorithm 1:** Window-Based Tensor Construction and Label Alignment

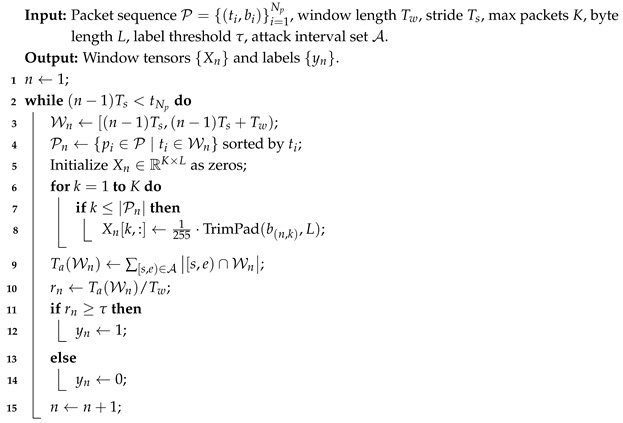



### 3.2. LDoS Pulse Characteristics from an Entropy Perspective

The key characteristic of LDoS attacks lies in their low average traffic rate combined with periodic pulses that trigger congestion or resource exhaustion at critical moments, thereby significantly degrading the throughput or availability of legitimate services. This property makes detection methods based solely on traffic magnitude or simple statistical thresholds ineffective, and also limits the ability of conventional mean-based attention mechanisms to highlight attack signals.

The LDoS process can be abstracted as a periodic square-wave pulse function [45]. Let the attack period be *T*, the pulse duration be $L_{p}$, and the peak rate be $R_{\mathrm{peak}}$. The attack traffic A(t) can be expressed as

(4) $$A\left(t\right)=\left\{\begin{array}{c} R_{\mathrm{peak}},\;kT\leq t<kT+L_{p}\\ 0,\;kT+L_{p}\leq t<\left(k+1\right)T \end{array}\right.,$$

where $k\in Z_{\geq 0}$ denotes the period index. The corresponding average rate is

(5) $$\bar{R}=R_{\mathrm{peak}}\cdot \frac{L_{p}}{T}.$$

In typical LDoS configurations, $L_{p}\ll T$, resulting in an average rate $\bar{R}$ that is significantly lower than the detection thresholds used for flooding attacks. The observed mixed traffic X(t) can be modeled as the superposition of background traffic B(t) and attack traffic A(t):

(6) X(t)=B(t)+A(t).

Since B(t) itself exhibits burstiness and strong noise, amplitude-based detection in the time domain cannot reliably distinguish the presence of A(t).

An information-theoretic perspective provides a more suitable basis for LDoS detection. During attack pulses, LDoS traffic often exhibits increased regularity and repetition, such as periodic convergence of packet inter-arrival times, repeated packet-length and flag patterns, and recurrent protocol fields or payload fragments. Such regularity alters the concentration of feature distributions, producing more pronounced differences in the entropy domain. Compared with Shannon entropy, Rényi entropy introduces a parameter α to control sensitivity to different regions of the distribution. When α>1, Rényi entropy places more emphasis on high-probability events, making it well suited for capturing distributional concentration caused by repetitive structures. For a discrete probability distribution $P={\left\{p_{i}\right\}}_{i=1}^{M}$, Rényi entropy is defined as

(7) $$H_{\alpha }\left(P\right)=\frac{1}{1-\alpha }\log\left(\sum_{i=1}^{M}p_{i}^{\alpha }\right),\;\alpha >0,\;\alpha \neq 1.$$

As α→1, $H_{\alpha }$ converges to Shannon entropy. In practice, α=2 is commonly adopted to obtain the collision entropy form, which is more sensitive to probability mass concentration and thus effective at amplifying repetitive structures during LDoS pulses.

Based on this motivation, the core idea of this work is to introduce differentiable Rényi entropy computation into the hidden feature space of deep networks and use entropy as a learnable attention-driving signal. This design enables the model to adaptively focus on entropy-anomalous feature channels and temporal segments under low-amplitude conditions. With window-based slicing, entropy-domain differences can be stably observed at both window and cross-window levels. Combined with dilated convolutions in TCNs, the model can capture periodic structures and long-range dependencies, enabling robust detection and early identification of LDoS attacks.

### 3.3. Overall Architecture of DELP-Net

**Figure 1:**
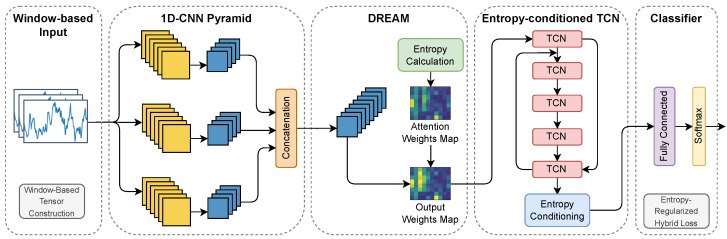
Overall architecture of DELP-Net.

DELP-Net is proposed as an end-to-end learning framework for LDoS detection, and its overall architecture is illustrated in Figure 1. The model takes windowed raw byte tensors as input and sequentially applies a multi-scale 1D-CNN pyramid, a differentiable Rényi entropy attention module (DREAM), an entropy-conditioned temporal convolutional network (TCN), and a classification head to produce window-level attack predictions. The architecture emphasizes lightweight design and online inference efficiency, relying primarily on convolutional operations and avoiding heavy self-attention backbones.

Let the input of the *n*-th time window be $X_{n}\in R^{K\times L}$. To explicitly model cross-window periodic dependencies, a window segment of length *M* is defined as

(8) $$X_{n}=\left\{X_{n-M+1},X_{n-M+2},\ldots ,X_{n}\right\}.$$

DELP-Net learns a mapping from $X_{n}$ to the predicted label ${\hat{y}}_{n}$. The overall forward computation can be summarized as

(9) $${\hat{y}}_{n}=f_{\theta }\left(X_{n}\right)=\mathrm{Cls}\left({\mathrm{TCN}}_{\omega }\left(\mathrm{Fuse}\left({\mathrm{DREAM}}_{\psi }\left({\mathrm{PyrCNN}}_{\phi }\left(X_{n}\right)\right)\right)\right)\right),$$

where ${\mathrm{PyrCNN}}_{\phi }$ denotes multi-scale 1D-CNN pyramid feature extraction, ${\mathrm{DREAM}}_{\psi }$ denotes differentiable Rényi entropy attention, Fuse represents multi-scale entropy-aware fusion, ${\mathrm{TCN}}_{\omega }$ denotes entropy-conditioned temporal modeling, and Cls is the classification head. The parameter set θ={ϕ,ψ,ω} contains all learnable parameters.

In practice, DELP-Net adopts a streaming window-level inference strategy. Each time a new window $X_{n}$ becomes available, the model uses $X_{n}$ to generate ${\hat{y}}_{n}$, enabling continuous detection and early warning. Since LDoS attacks typically exhibit periodic pulse behavior, the segment length *M* together with the receptive field of the TCN determines the model’s ability to capture such periodic structures.

### 3.4. Multi-Scale 1D-CNN Pyramid for Window Feature Extraction

The multi-scale 1D-CNN pyramid module is designed to extract hierarchical representations from raw byte sequences within each time window, covering multiple receptive-field scales to accommodate the multi-scale nature of network traffic patterns. This module adopts convolution as the core operator, resulting in low computational complexity and suitability for online deployment. To preserve the temporal order of the input structure, the window tensor $X_{n}$ is serialized according to packet order to form a one-dimensional sequence,

(10) $$s_{n}=\mathrm{vec}\left(X_{n}\right)\in R^{S},\;S=K\cdot L,$$

where vec(·) denotes row-wise vectorization. This representation preserves protocol field ordering and payload fragment continuity at the byte level while maintaining the relative positional relationships among packets through sequential concatenation.

The pyramid architecture adopts parallel multi-branch convolutions, inspired by the Inception design [46]. Let the branch set be $R=\{r_{1},r_{2},r_{3}\}$, corresponding to different kernel sizes, with a typical configuration of $r_{1}=3$, $r_{2}=5$, and $r_{3}=11$. Each branch consists of a Conv1D layer followed by batch normalization, ReLU activation, and downsampling pooling, producing branch-level feature maps

(11) $$F_{n}^{\left(r\right)}=\mathrm{Pool}\left(\mathrm{ReLU}(\mathrm{BN}\left(\mathrm{Conv}1D\left(s_{n};W_{r}\right)\right))\right),$$

where $W_{r}$ denotes the convolution parameters with kernel size *r*, and Pool represents average pooling. The outputs from different scales are concatenated along the channel dimension to form the fused feature map $F_{n}$, which serves as the input to the subsequent DREAM entropy-attention module:

(12) $$F_{n}=\mathrm{Concat}\left(F_{n}^{\left(r_{1}\right)},F_{n}^{\left(r_{2}\right)},F_{n}^{\left(r_{3}\right)}\right).$$

The design of multi-scale convolution kernels has clear security semantics. Small-scale kernels are effective at capturing local byte patterns, which often correspond to localized combinations of protocol fields. Medium-scale kernels are more suitable for modeling structured fragments of protocol headers. Large-scale kernels cover longer contiguous byte ranges, facilitating the capture of repeated payload fragments and periodic traces formed by cross-packet concatenation within a window. Compared with single-scale convolution, the pyramid structure enables simultaneous extraction of fine-grained and coarse-grained traffic fingerprints, providing more discriminative candidate channels for subsequent entropy-based attention.

To control model size and maintain a lightweight design, several strategies are adopted. First, the number of pyramid stages is kept small, and stable performance gains can typically be achieved with two to three stacked stages. Second, channel widths across branches are balanced, and redundant channels are reduced to mitigate overfitting. Third, grouped convolution or depthwise separable convolution can be optionally employed to replace standard convolution, further reducing parameter count and FLOPs, which facilitates model compression and practical deployment.

### 3.5. Differentiable Rényi Entropy Attention Mechanism (DREAM)

**Figure 2:**
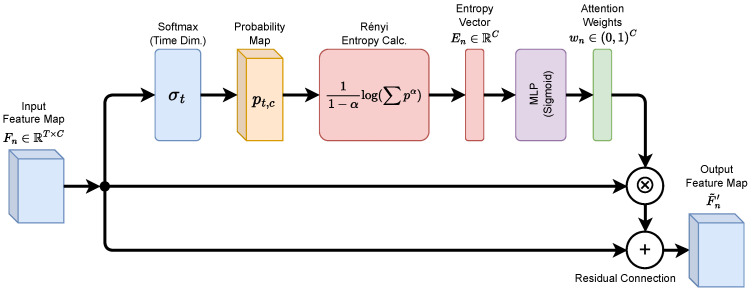
Architecture of the DREAM module.

The DREAM module introduces a differentiable Rényi entropy operator into the feature space and maps entropy values to attention weights for channel-wise recalibration of pyramid features. The structure of DREAM is illustrated in Figure 2. Unlike attention mechanisms based on mean or activation magnitude statistics, DREAM emphasizes distributional concentration, making it particularly suitable for characterizing the regularity and repetition exhibited by LDoS traffic under low-amplitude conditions.

Let the window-level feature map output by the multi-scale 1D-CNN pyramid be denoted as $F_{n}\in R^{T\times C}$, where *T* is the sequence length and *C* is the number of channels. The activation value at position *t* and channel *c* is given by $F_{n}\left[t,c\right]$. Here, the feature map $F_{n}$ represents the intermediate representation extracted by the multi-scale convolutional pyramid from the raw traffic window $X_{n}$. Each channel corresponds to a specific response pattern learned from byte-level traffic structures, while the sequence dimension preserves the temporal ordering of packets within the window. Therefore, the feature tensor $F_{n}$ jointly encodes protocol-field structures, packet-level byte patterns, and intra-window temporal relationships.

To compute differentiable entropy, a pseudo-probability distribution is first constructed from continuous features. Specifically, softmax normalization is applied along the sequence dimension, mapping each channel’s energy distribution into a probability vector:

(13) $$p_{t,c}=\frac{\exp(F_{n}\left[t,c\right])}{\sum_{j=1}^{T}\exp\left(F_{n}\left[j,c\right]\right)},\;t=1,2,\ldots ,T.$$

This yields the channel-wise distribution $P_{c}={\left\{p_{t,c}\right\}}_{t=1}^{T}$, satisfying $\sum_{t=1}^{T}p_{t,c}=1$. This construction relies only on exponential, summation, and division operations, ensuring differentiability and computational efficiency suitable for lightweight online detection.

Rényi entropy is then computed on $P_{c}$ to obtain the channel-level entropy vector $E_{n}\in R^{C}$, with the *c*-th component defined as

(14) $$E_{n}\left[c\right]=\frac{1}{1-\alpha }\log\left(\sum_{t=1}^{T}p_{t,c}^{\alpha }\right),\;\alpha >0,\;\alpha \neq 1.$$

In this formulation, $p_{t,c}$ denotes the normalized activation probability at temporal position *t* for channel *c*, obtained through the softmax operation in Equation (13). The parameter α is the order of Rényi entropy, which controls the sensitivity of the entropy measure to concentration in the feature distribution. When α>1, the entropy becomes more sensitive to dominant activations, which helps highlight repetitive structures induced by LDoS traffic pulses.

In the main model, α=2 is adopted to emphasize sensitivity to high-probability concentration, thereby amplifying low-entropy phenomena induced by repetitive structures. Since both Equations (13) and (14) are composed entirely of differentiable operations such as exponential, summation, logarithm, and division, the entropy computation can be naturally incorporated into gradient-based optimization.

The entropy vector is mapped to channel weights $w_{n}\in {\left(0,1\right)}^{C}$ through a lightweight two-layer feedforward network:

(15) $$w_{n}=\sigma \left(W_{2}\cdot \mathrm{ReLU}\left(W_{1}\cdot E_{n}\right)\right),$$

where $W_{1}$ and $W_{2}$ are learnable parameters and σ(·) denotes the sigmoid function. Channel-wise recalibration is then applied to the feature map:

(16) $$F_{n}^{\prime }\left[t,c\right]=w_{n}\left[c\right]\cdot F_{n}\left[t,c\right].$$

This operation establishes a direct interaction between entropy statistics and feature representations. Channels with entropy patterns indicative of repetitive or concentrated activations receive higher attention weights, while channels dominated by irregular or noisy responses are suppressed. As a result, the network adaptively emphasizes feature channels that exhibit entropy anomalies consistent with LDoS traffic characteristics.

To improve training stability and preserve the original representation, a residual formulation is adopted:

(17) $${\tilde{F}}_{n}=F_{n}+F_{n}^{\prime }.$$

Through this process, DREAM dynamically suppresses irregular, high-entropy noise channels while enhancing the discriminative contribution of entropy-anomalous channels, enabling the model to more effectively capture weak LDoS signatures.

### 3.6. Entropy Pyramid Fusion

**Figure 3:**
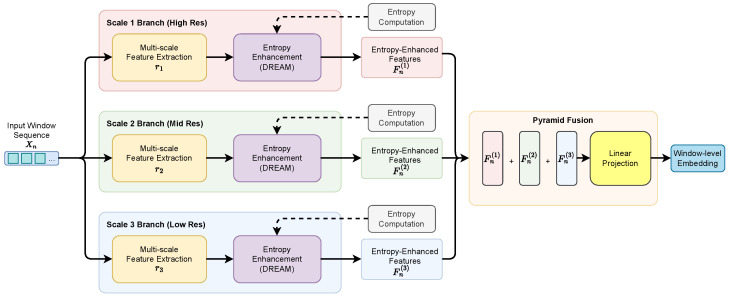
Entropy pyramid fusion across multi-scale branches with entropy-enhanced representations for window-level embedding (S = 3).

Multi-scale convolutional pyramids produce complementary features at different receptive-field scales, and LDoS periodic pulses may manifest distinct entropy anomalies across these scales. To enhance coverage of multi-scale regularities, an entropy pyramid representation is constructed and fused into a compact window-level embedding, which serves as the input to subsequent temporal modeling. An overview of this process is shown in Figure 3.

Let the pyramid consist of *S* scale outputs indexed by s=1,2,…,S, where the feature map at scale *s* is denoted as $F_{n}^{\left(s\right)}\in R^{T_{s}\times C_{s}}$. DREAM is independently applied at each scale to obtain enhanced features ${\tilde{F}}_{n}^{\left(s\right)}$ and entropy vectors $E_{n}^{\left(s\right)}$. To derive a fixed-dimensional representation at the window level, sequence-wise aggregation is applied to the enhanced features using global average pooling, yielding a scale-level vector $g_{n}^{\left(s\right)}\in R^{C_{s}}$:

(18) $$g_{n}^{\left(s\right)}\left[c\right]=\frac{1}{T_{s}}\sum_{t=1}^{T_{s}}{\tilde{F}}_{n}^{\left(s\right)}\left[t,c\right],\;c=1,2,\ldots ,C_{s}.$$

To explicitly inject entropy information, the scale-level entropy vector is normalized and concatenated with the corresponding feature vector, forming an entropy-enhanced representation:

(19) $$z_{n}^{\left(s\right)}=\mathrm{Concat}\left(g_{n}^{\left(s\right)},\;\mathrm{Norm}\left(E_{n}^{\left(s\right)}\right)\right),$$

where Norm(·) denotes linear normalization or standardization to ensure consistency of entropy magnitudes across scales. The representations from all scales are concatenated to form the window-level entropy pyramid vector:

(20) $$z_{n}=\mathrm{Concat}\left(z_{n}^{\left(1\right)},z_{n}^{\left(2\right)},\ldots ,z_{n}^{\left(S\right)}\right).$$

To control dimensionality and maintain a lightweight design, a linear projection is applied to compress $z_{n}$ into a fixed-dimensional embedding $h_{n}\in R^{d}$:

(21) $$h_{n}=W_{f}z_{n}+b_{f},$$

where $W_{f}$ and $b_{f}$ are learnable parameters. The resulting vector $h_{n}$ serves as the window-level fused representation for subsequent TCN-based temporal modeling.

Considering the strong cross-window regularity of LDoS attacks, entropy variation trends also provide valuable discriminative information. Entropy difference features are further evaluated:

(22) $$\Delta E_{n}^{\left(s\right)}=E_{n}^{\left(s\right)}-E_{n-1}^{\left(s\right)}.$$

This feature measures entropy change between adjacent windows and can be concatenated with $E_{n}^{\left(s\right)}$ during fusion to enhance sensitivity to pulse boundaries. Since $\Delta E_{n}^{\left(s\right)}$ involves only difference operations, it introduces negligible additional inference overhead.

### 3.7. Temporal Modeling with Entropy-Conditioned TCN

The key behavioral characteristic of LDoS attacks is manifested in cross-window periodic pulses and long-range temporal dependencies. Relying solely on within-window features is insufficient to stably capture such rhythmic structures. To address this issue, a lightweight Temporal Convolutional Network (TCN) is employed to model sequences of window-level representations. By using dilated convolutions, TCN expands the receptive field without a significant increase in parameter count, making it suitable for periodic pattern modeling in online detection scenarios.

Let the window-level representation obtained from entropy pyramid fusion be denoted as $h_{n}\in R^{d}$. A sequence of *M* consecutive windows is constructed as the input to the temporal model:

(23) $$H_{n}=\left\{h_{n-M+1},h_{n-M+2},\ldots ,h_{n}\right\}.$$

The TCN consists of $L_{t}$ stacked layers of one-dimensional dilated convolutions. For the *ℓ*-th layer, the dilation rate is denoted as $\delta_{\ell }$ and the kernel size is $k_{t}$. For the *i*-th position in the sequence, the dilated convolution can be written as

(24) $$u_{i}^{\left(\ell \right)}=\sum_{j=0}^{k_{t}-1}W_{j}^{\left(\ell \right)}\cdot u_{i-j\delta_{\ell }}^{\left(\ell -1\right)}+b^{\left(\ell \right)},$$

where $u_{i}^{\left(0\right)}=h_{n-M+i}$ denotes the input feature vector at position *i*, and $W_{j}^{\left(\ell \right)}$ and $b^{\left(\ell \right)}$ are learnable parameters. Exponentially increasing dilation rates $\delta_{\ell }=2^{\ell -1}$ are adopted to rapidly enlarge the receptive field, allowing the model to cover multiple LDoS attack periods. Residual connections, together with normalization and ReLU activation, are applied to improve training stability and mitigate gradient vanishing.

To explicitly inject entropy information into the temporal modeling process, an entropy-conditioned mechanism is introduced to modulate the channel responses of the TCN. Let the entropy condition vector corresponding to window *n* be denoted as $e_{n}\in R^{d_{e}}$. This vector can be constructed by concatenating entropy statistics from the entropy pyramid stage or extracted as a normalized entropy subvector during fusion. A sequence $E_{n}\;=\;\left\{e_{n-M+1},\ldots ,e_{n}\right\}$ is formed and linearly mapped to generate gating coefficients:

(25) $$g_{i}=\sigma \left(W_{g}e_{n-M+i}+b_{g}\right),$$

where $g_{i}\in {\left(0,1\right)}^{d_{g}}$ denotes the gating vector, and $W_{g}$ and $b_{g}$ are learnable parameters. For an intermediate TCN representation ${\hat{u}}_{i}^{\left(\ell \right)}$, channel-wise modulation is applied as

(26) $${\tilde{u}}_{i}^{\left(\ell \right)}=g_{i}⊙{\hat{u}}_{i}^{\left(\ell \right)},$$

where ⊙ denotes the Hadamard product. This mechanism enables the model to place greater emphasis on windows exhibiting entropy anomalies or abrupt entropy changes, thereby enhancing sensitivity to LDoS pulses under low signal-to-noise conditions. Compared with incorporating self-attention backbones, entropy-conditioned TCN introduces only a small number of additional linear parameters, maintaining a lightweight and deployment-friendly design.

After $L_{t}$ TCN layers, the output sequence ${\left\{u_{i}^{\left(L_{t}\right)}\right\}}_{i=1}^{M}$ is obtained. A tail-aggregation strategy is adopted to derive the temporal representation for the current window *n*:

(27) $$r_{n}=u_{M}^{\left(L_{t}\right)}.$$

This representation is fed into the classification head to produce the final prediction. Tail aggregation is consistent with the sliding-window inference strategy and facilitates real-time alert generation in online systems.

### 3.8. Classification with Entropy-Regularized Hybrid Loss

Based on the temporal representation $r_{n}$, a lightweight classification head is employed to output the window-level prediction probability. The classifier consists of fully connected layers with ReLU activation and dropout to reduce overfitting. Let the classifier parameters be denoted as $\Theta_{c}$. For the binary classification case, the output is given by

(28) $${\hat{y}}_{n}=\mathrm{Softmax}\left(W_{c}r_{n}+b_{c}\right),$$

where ${\hat{y}}_{n}\in R^{2}$, and $W_{c}$ and $b_{c}$ are learnable parameters. The formulation can be directly extended to multi-class scenarios by increasing the output dimension to the number of classes $C_{y}$.

An entropy-regularized hybrid loss is adopted to enhance class separability in the entropy domain and improve robustness under complex background traffic. The base classification loss is defined as the cross-entropy loss:

(29) $$L_{\mathrm{CE}}=-\frac{1}{N}\sum_{n=1}^{N}\sum_{c=1}^{C_{y}}1\left[y_{n}=c\right]\log\left({\hat{y}}_{n,c}\right),$$

where *N* denotes the number of training samples, $C_{y}$ is the number of classes, 1[·] is the indicator function, and ${\hat{y}}_{n,c}$ is the predicted probability for class *c*.

The entropy regularization term is applied to the channel entropy vectors produced by DREAM to directly constrain the distributional properties of hidden representations. Let $E_{n}$ denote the set of entropy vectors obtained from selected scales. For simplicity, these vectors are aggregated into a scalar entropy statistic:

(30) $${\bar{E}}_{n}=\frac{1}{|E_{n}|}\sum_{E\in E_{n}}\frac{1}{\dim(E)}\sum_{c=1}^{\dim(E)}E\left[c\right].$$

A class-conditional entropy constraint is adopted to encourage stronger concentration for attack samples while avoiding overly restrictive assumptions on benign traffic. The entropy regularization loss is defined as

(31) $$L_{\mathrm{ER}}=\frac{1}{N}\sum_{n=1}^{N}y_{n}\cdot {\bar{E}}_{n},$$

where $y_{n}\in \left\{0,1\right\}$ denotes the binary label, with $y_{n}=1$ indicating an attack window. Minimizing $L_{\mathrm{ER}}$ encourages lower average entropy for attack samples, reinforcing the concentration of repetitive structures in the feature space. This constraint aligns with the sensitivity of Rényi entropy to high-probability concentration and contributes to improved detection performance for LDoS traffic.

The overall training objective is defined as

(32) $$L_{\mathrm{Total}}=L_{\mathrm{CE}}+\lambda L_{\mathrm{ER}},$$

where λ is a weighting coefficient. The model parameters are optimized by minimizing $L_{\mathrm{Total}}$ using stochastic gradient-based optimizers. To avoid representation collapse caused by excessive entropy regularization, a relatively small λ is adopted and tuned on the validation set. Dropout is also applied in the DREAM and TCN modules to further improve generalization. The impact of entropy regularization on the false-positive rate and robustness is analyzed in the Section 4.

Algorithm 2 summarizes the end-to-end training process of DELP-Net, including multi-scale feature extraction, differentiable Rényi entropy attention, entropy pyramid fusion, entropy-conditioned TCN temporal modeling, and optimization with the entropy-regularized hybrid loss.

**Algorithm 2:** End-To-End Training Process of DELP-Net

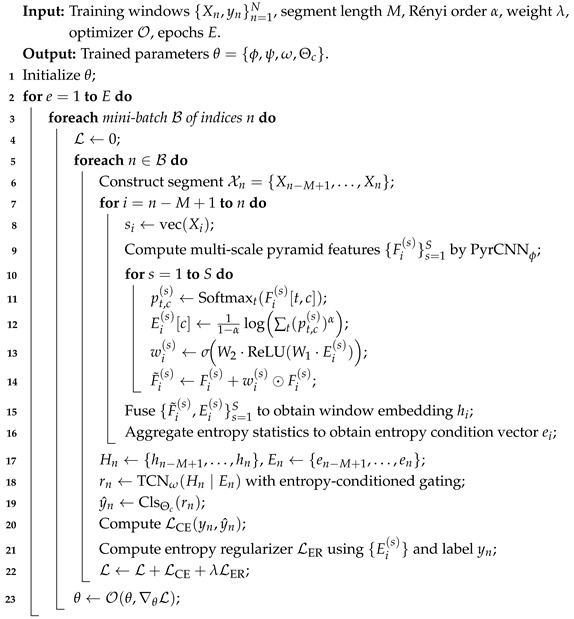



## 4. Experiments

### 4.1. Datasets and Sample Construction

#### 4.1.1. Datasets

To closely reflect practical intrusion detection scenarios, this study constructs the evaluation benchmark by combining publicly available datasets with traffic collected from a self-built experimental platform.

Three categories of data sources are used in this work. The first is the CICIDS2017 dataset [47], which contains a wide range of benign traffic and representative DoS-related attack traffic, together with relatively complete flow-level and packet-level annotations. The second is the CICIDS2018 dataset, which is larger in scale and exhibits stronger diversity in background traffic, enabling evaluation of model generalization under complex noise conditions [48]. The third is an extended low-rate DoS dataset collected on a self-built traffic generation and capture platform. This dataset incorporates more realistic characteristics such as link jitter and service-level traffic fluctuations, and better reflects practical deployment environments [49].

All data sources include benign traffic as the background class. The low-rate DoS attack types evaluated in this study mainly include Slowloris, Slow POST, Slow Read, Pwnloris, Torshammer, and Httpbog. These six attack categories are selected because they represent typical low-rate DoS behaviors and are consistently observable across the public and self-collected data sources used in this work.

For the two public datasets, traffic samples are extracted from the original traffic records, while benign samples are collected from normal background traffic sessions provided in the datasets. CICIDS2017 contains protocol-diverse traffic including TCP, UDP, and ICMP, whereas CICIDS2018 mainly contains TCP and UDP traffic under more complex background conditions. After filtering and sample construction, the subset used in this study contains approximately 62,000 samples from CICIDS2017 and 71,000 samples from CICIDS2018. For CICIDS2017, about 31,500 samples are benign and 30,500 samples are attack samples. For CICIDS2018, about 36,200 samples are benign and 34,800 samples are attack samples.

The self-built dataset is collected in a controlled experimental environment consisting of a traffic generation host, a target web service, and a monitoring node used for packet capture. Benign traffic is generated through common service access behaviors such as web browsing, file transfers, and streaming-like HTTP sessions, so the collected background traffic contains realistic application-level fluctuations. Low-rate DoS traffic is then injected by launching Slowloris, Slow POST, Slow Read, Pwnloris, Torshammer, and Httpbog attacks against the target service using publicly available tools. During the collection process, network delay perturbation and traffic jitter are intentionally introduced at the link and application levels to simulate unstable service access conditions that may appear in practice.

The self-collected dataset mainly consists of TCP traffic and contains approximately 28,500 samples, including about 14,800 benign samples and 13,700 attack samples. The total traffic collection duration is approximately 4 h, and the raw packet traces are captured continuously during both benign-only and attack-injection periods. Labels are assigned according to the known attack injection schedule and then verified through timestamp alignment between attack launch logs and captured traffic records. In this way, each traffic sample can be associated with a clear benign or malicious label, which improves the reliability of the annotation process.

Table 1 summarizes the main statistics of the datasets used in our experiments, including the number of samples, benign and attack distribution, protocol types, and training/testing splits.

To ensure fair comparison across different data sources, all datasets are processed using the same preprocessing and sample construction pipeline. Traffic flows are segmented into fixed-length traffic samples and normalized before being fed into the model. For each dataset, the training and testing sets are divided using a 70/30 split. The datasets are split chronologically at the flow level before window segmentation in order to avoid potential data leakage between training and testing samples.

#### 4.1.2. Sample Construction

To match the window-level end-to-end input representation described in Section 3, the original PCAP files are parsed into time-ordered packet sequences and first aggregated into flows based on the five-tuple [50]. Flow aggregation is mainly used for label alignment and to prevent window concatenation across different sessions. Each flow is then segmented into fixed-length sliding time windows, and each window corresponds to one sample.

For the raw byte input within each window, at most *K* packets are selected in chronological order. Each packet is truncated or zero-padded to *L* bytes, forming a fixed-shape tensor $X\in R^{K\times L}$. This construction preserves protocol header fields and partial payload fragments at the byte level while explicitly aligning with the periodic pulse behavior of LDoS attacks through time-window slicing.

### 4.2. Evaluation Metrics

This study adopts Accuracy, DetectionRate, FalsePositiveRate, Precision, and F1 score as evaluation metrics. To be consistent with common reporting practices in the intrusion detection literature, all metrics except for F1 are reported in percentage form. Let TP, TN, FP, and FN denote true positives, true negatives, false positives, and false negatives, respectively. The metrics are defined as follows:

(33) $$\left\{\begin{array}{c} Accuracy\;\left(ACC\right)=\frac{TP+TN}{TP+TN+FP+FN}\\ Detection\;Rate\;\left(DR\right)=\frac{TP}{TP+FN}\\ False\;Positive\;Rate\;\left(FPR\right)=\frac{FP}{FP+TN}\\ Precision\;\left(Pre\right)=\frac{TP}{TP+FP}\\ F1=\frac{2\cdot Pre\cdot DR}{Pre+DR} \end{array}\right.$$

### 4.3. Window-Based Preprocessing and Data Splits

A sliding time window is used as the basic sampling unit. The window length is set to $T_{w}$ and the stride is set to $T_{s}$. Within each window, at most *K* packets are selected in chronological order, and each packet is truncated or zero-padded to *L* bytes, yielding a fixed-shape input tensor $X\in R^{K\times L}$. If the number of packets in a window is smaller than *K*, the remaining rows are padded with zeros. If a window contains far fewer than *K* packets and remains largely idle, it may lack sufficient discriminative information. To control the proportion of such low-information windows, we discard windows whose packet count is lower than $K_{\min}$ during data construction, where $K_{\min}$ is selected on the validation set.

For temporal modeling, adjacent windows within the same flow are grouped into an ordered segment of length *M* as the input to the TCN. If a flow is too short to form *M* consecutive windows, it is discarded to ensure consistency for sequence modeling. Window labels are generated from original annotations and attack intervals: if the overlap ratio between a window and an attack interval exceeds the threshold τ, the window is labeled as an attack window; otherwise, it is labeled as benign. The preprocessing parameters used are summarized in Table 2.

### 4.4. Baselines

We consider the following classic baselines, covering statistical detection, traditional machine learning, and deep learning models. All baselines are trained and evaluated under the same window-based preprocessing configuration as DELP-Net.

- **Entropy-Threshold**: A window-level entropy threshold detector. For each time window, empirical distributions are constructed on discretized traffic attributes. Rényi entropy statistics are computed and compared against thresholds estimated from benign windows in the training set and tuned on the validation set to control false alarms.
- **RF**: A Random Forest classifier trained on handcrafted window-level statistical features. The feature vector includes byte counts, mean and variance of inter-arrival times, simple directional statistics within the window, TCP flag counts, and packet-length quantiles. Hyperparameters are selected on the validation set.
- **XGBoost**: A gradient-boosted decision tree classifier trained on the same handcrafted window-level feature set as RF. Model hyperparameters are tuned on the validation set to ensure a fair comparison.
- **1D-CNN**: An end-to-end convolutional baseline operating on raw bytes. Each window is represented as a fixed-shape K×L byte tensor (with truncation/padding) and then serialized as a 1D sequence. Stacked single-scale Conv1D layers extract window representations, followed by a fully connected classification head.
- **LSTM**: A deep sequence baseline for raw-window modeling. The K×L byte tensor is treated as an ordered sequence, and an LSTM encoder is used to model temporal dependencies within the window. The final hidden state is fed into a fully connected classifier.

### 4.5. Implementation Details

All experiments are performed on a workstation featuring an Intel Xeon(R) Platinum 8352V processor operating at 2.1 GHz, 64 GB of system memory, and an NVIDIA RTX 4090D graphics card with 24 GB of video memory. The operating system is Ubuntu 22.04 LTS. All models are implemented using PyTorch 2.3.0, and GPU acceleration is enabled via CUDA 12.1 and cuDNN 8.

During training, the Adam optimizer is used with an initial learning rate of $1\times 10^{-3}$, and the learning rate is decayed when the validation performance no longer improves. To enhance generalization, dropout is applied to convolutional layers and fully connected layers, with the dropout rate selected on the validation set. In the DREAM module, the Rényi order is fixed to α=2, and the entropy-regularization weight λ is tuned on the validation set. Early stopping is adopted for all models: if the validation F1 score does not improve for a number of consecutive epochs, training is terminated and the best-performing checkpoint is restored. The key hyperparameters used in the final configuration are summarized in Table 3.

### 4.6. Detection Performance

This subsection reports the main detection results of DELP-Net for LDoS attack subclasses. To highlight the model’s adaptability to different LDoS patterns, we evaluate window-level detection performance for six attack types: Slowloris, Slow POST, Slow Read, Pwnloris, Torshammer, and Httpbog. The per-attack results of the proposed method are presented in Table 4. DELP-Net consistently achieves high detection rates and high precision across all subclasses, with an average F1 score of 0.9877. These results indicate that the proposed method effectively captures different weak regularity patterns of LDoS traffic.

Table 5 reports window-based comparison results against representative classical baselines, including an entropy-threshold detector, traditional machine learning classifiers, and commonly used deep sequence models. As can be observed, the entropy-threshold baseline suffers from a relatively high false-positive rate, indicating that fixed-threshold statistics are easily perturbed by bursty and diverse benign traffic. Traditional machine learning methods (RF and XGBoost) further show limited discriminative capability under the raw-traffic window setting, resulting in both lower DR and higher FPR. End-to-end deep baselines (1D-CNN and LSTM) improve overall performance but still exhibit non-negligible false alarms, suggesting that modeling raw bytes alone without explicitly leveraging distributional regularity is insufficient for robust low-rate DoS detection.

In contrast, DELP-Net achieves the best trade-off across all key metrics, reaching a DR of 98.69% and an F1 score of 0.9877 while reducing the false-positive rate to 1.15%. The consistent gains in both DR (98.69%) and Precision (98.85%) indicate that the proposed entropy-driven representation learning and temporal modeling effectively capture the weak yet regular patterns of LDoS traffic, thereby improving detection robustness under complex benign background dynamics.

## 5. Discussion

### 5.1. Comparison with Recent State-of-the-Art Methods

To further assess the effectiveness of the proposed DELP-Net, we compare it with several recent state-of-the-art detection methods reported in the literature. These methods span different detection paradigms, including feature-engineered classifiers, signal- or image-based representations, hybrid learning frameworks, and deep neural network models.

As shown in Table 6, DELP-Net achieves the best overall performance among the compared methods, particularly in terms of F1 score and detection rate. Compared with earlier approaches that rely on handcrafted features or fixed representations, the proposed model benefits from end-to-end learning directly on raw traffic windows, which helps preserve fine-grained protocol and temporal information.

Recent deep learning-based methods, such as MSCBL-AND and ERT-EDR, demonstrate competitive results by leveraging multi-scale representations or ensemble decision strategies. In contrast, DELP-Net explicitly embeds differentiable Rényi entropy into the network architecture, enabling the model to capture distributional regularity and repetition patterns that are characteristic of low-rate DoS attacks but are often overlooked by amplitude- or energy-driven mechanisms.

Moreover, unlike methods that depend on complex preprocessing pipelines or high-dimensional handcrafted features, DELP-Net adopts a lightweight convolutional backbone and supports window-level online inference. This design allows the model to achieve superior detection performance while remaining suitable for practical deployment scenarios with strict latency and throughput constraints.

### 5.2. Ablation Studies

We evaluate the contribution of each major component in DELP-Net under the same window-based preprocessing and split strategy as in Section 4. We report ACC, DR, FPR, Precision, and F1 to reflect the trade-off between detection performance and false alarms.

#### 5.2.1. Ablation Settings

To quantify the effect of each component, we construct the following variants by removing or replacing specific modules while keeping all other settings unchanged.

- **w/o DREAM**: Remove DREAM and disable entropy-driven channel recalibration, and pyramid features are forwarded directly to subsequent modules.
- **DREAM to SE**: Replace DREAM with the Squeeze-and-Excitation (SE) channel attention [14].
- **DREAM to CBAM**: Replace DREAM with CBAM using mean-based channel and spatial attention [57].
- **w/o Entropy Pyramid Fusion**: Keep multi-scale convolution and DREAM, but remove multi-scale entropy fusion and use only the final-scale feature as the window representation.
- **w/o TCN**: Remove the temporal modeling module, and window embeddings are fed directly into the classifier.
- **TCN w/o Entropy Conditioning**: Keep TCN but disable entropy-conditioned gating, and temporal modeling uses window embeddings only.
- **DELP-Net (Full)**: The full model.

#### 5.2.2. Ablation Results

Table 7 summarizes the ablation results. Removing DREAM causes a clear degradation, with F1 dropping to 0.9439 and FPR increasing to 5.48%, indicating that entropy-driven channel recalibration is critical for suppressing false alarms in low-rate settings. Replacing DREAM with SE improves performance (F1 = 0.9585), while CBAM yields further gains (F1 = 0.9656), showing that conventional attention helps but remains less effective than entropy-driven attention for capturing LDoS regularity. Disabling entropy pyramid fusion reduces F1 to 0.9670, suggesting that aggregating entropy-aware representations across multiple receptive-field scales improves coverage of diverse LDoS patterns. Removing the TCN decreases F1 to 0.9761, confirming the importance of modeling cross-window temporal dependencies. Moreover, disabling entropy-conditioned gating within TCN further reduces F1 to 0.9820, highlighting the complementary role of entropy information in guiding temporal decision-making.

Overall, DELP-Net achieves the best performance, validating the effectiveness of DREAM, entropy pyramid fusion, and entropy-conditioned temporal modeling.

### 5.3. Sensitivity Analysis of Rényi Parameter α

This subsection investigates the sensitivity of DELP-Net to the Rényi order α used in the DREAM module. Since α controls how strongly the entropy measure emphasizes high-probability events, it directly affects how DREAM responds to distributional concentration and repetitive patterns, which are key characteristics of low-rate DoS traffic. Intuitively, a larger α assigns more weight to dominant activations, whereas a smaller α increases the contribution of low-probability components, potentially weakening the contrast between benign burstiness and attack-induced regularity.

#### 5.3.1. Experimental Setting

We keep the network architecture, window-based preprocessing, training schedule, and data splits unchanged, and vary only the Rényi order in DREAM. Specifically, we evaluate α∈{0.5,1,2,3,4,5}. All settings share the same hyperparameters and early-stopping strategy to ensure a fair comparison.

#### 5.3.2. Results and Discussion

**Figure 4:**
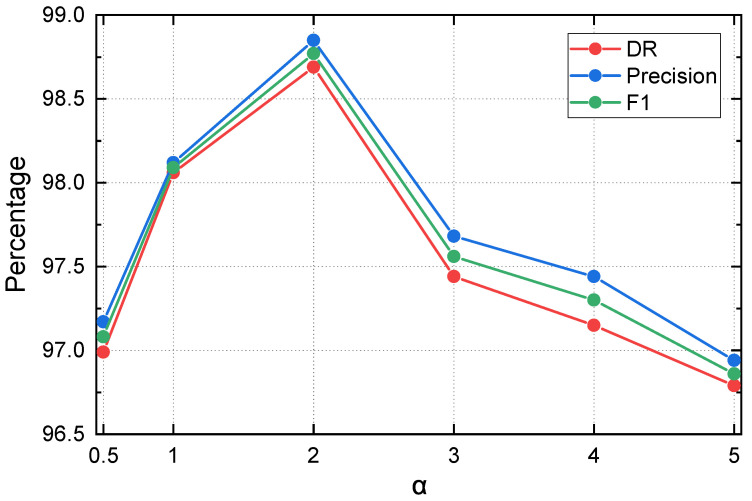
Sensitivity analysis of the Rényi order α in DREAM.

Figure 4 summarizes the performance trends under different α values. The best overall performance is achieved at α=2. When α deviates from 2, all key metrics degrade to varying degrees, with a consistent drop in F1. For example, using Shannon entropy (α=1) yields slightly lower performance, while smaller α (0.5) further reduces F1 to 0.9708. For larger α values, the performance also decreases, suggesting that overly emphasizing a small set of dominant activations may reduce robustness under background fluctuations.

From a mathematical perspective, α controls how strongly the Rényi entropy emphasizes different regions of the feature distribution. When α<1, relatively greater importance is assigned to low-probability components, making the entropy more sensitive to weak background fluctuations and minor irregularities. As a result, the contrast between benign burstiness and attack-induced concentration may be weakened, which can reduce detection sensitivity. In contrast, when α>1, the entropy places more emphasis on dominant probability mass, thereby making the model more sensitive to concentrated and repetitive activation patterns that are characteristic of LDoS attacks. However, if α becomes excessively large, the entropy may over-focus on only a few dominant activations, which can reduce robustness to normal traffic variation and lead to slight performance degradation.

Overall, α=2 provides the most favorable balance between detection capability and false-alarm suppression. This observation is consistent with the collision-entropy-like behavior at α=2, which effectively highlights concentration caused by repetitive attack patterns while avoiding excessive sensitivity that may arise with very large α. Therefore, we adopt α=2 as the default configuration for DREAM in all main experiments.

### 5.4. Robustness to Attack Parameter Variations

Low-rate DoS attacks can be easily adapted by adversaries through manipulating key pulse parameters, such as the pulse period *T*, pulse duration $L_{p}$, and peak rate $R_{\mathrm{peak}}$, and by injecting timing jitter to reduce regularity. Therefore, beyond in-distribution evaluation, it is important to quantify how stable DELP-Net remains under controlled parameter perturbations that alter the temporal dynamics while preserving the low-rate nature of the attack.

#### 5.4.1. Experimental Setting

We construct perturbed test sets by modifying one or more pulse parameters in the LDoS process, following the pulse abstraction in Equation (4). Specifically, we consider four settings: (i) *None*, the default evaluation configuration; (ii) *Low (in-distribution)*, where parameters remain close to the training distribution with a small amount of noise; (iii) *Medium*, where *T* and $L_{p}$ are varied to change the duty cycle and periodicity while keeping the overall behavior comparable; and (iv) *High*, where *T*, $L_{p}$, and $R_{\mathrm{peak}}$ are jointly perturbed and additional timing jitter is introduced to weaken the periodic signature [58,59]. All other components are kept unchanged. We report DR, Precision, and F1 under the same window-based protocol as the main experiments.

#### 5.4.2. Results and Discussion

**Figure 5:**
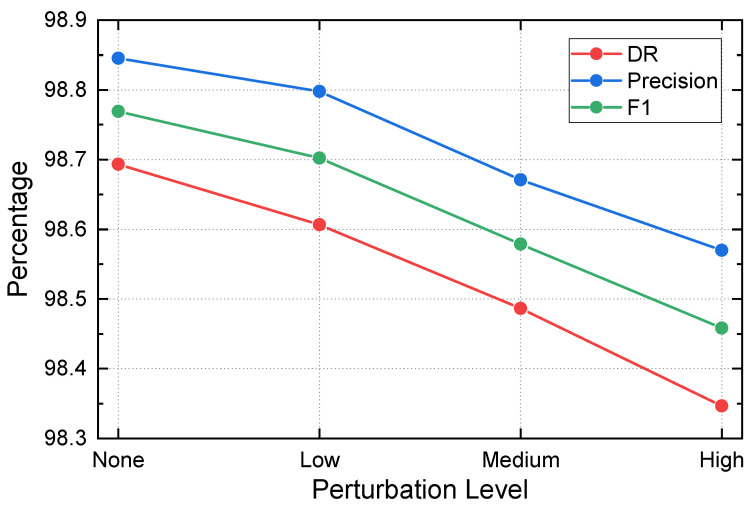
Robustness of DELP-Net under LDoS attack parameter variations. The perturbation levels correspond to modifying *T*, $L_{p}$, and $R_{\mathrm{peak}}$.

Figure 5 summarizes the performance trends under different perturbation levels. DELP-Net maintains consistently strong performance as perturbation intensity increases. Starting from the unperturbed setting, the model exhibits only mild and monotonic degradation under Low and Medium perturbations, indicating that modest changes in *T* and $L_{p}$ do not substantially affect the learned detection cues. Even under the High perturbation setting, where multiple parameters are varied and jitter is injected, DELP-Net retains a high detection rate and low false-positive rate.

These results suggest that the proposed entropy-driven representation learning is not overly dependent on a single fixed pulse configuration. Instead, DELP-Net captures more stable distributional regularity and cross-window temporal patterns that persist across a range of LDoS parameterizations. The limited decrease in F1 under stronger perturbations further indicates that the model does not simply overfit to narrow periodic signatures, but remains robust under adversarial timing variations.

### 5.5. Feature Space Visualization

**Figure 6:**
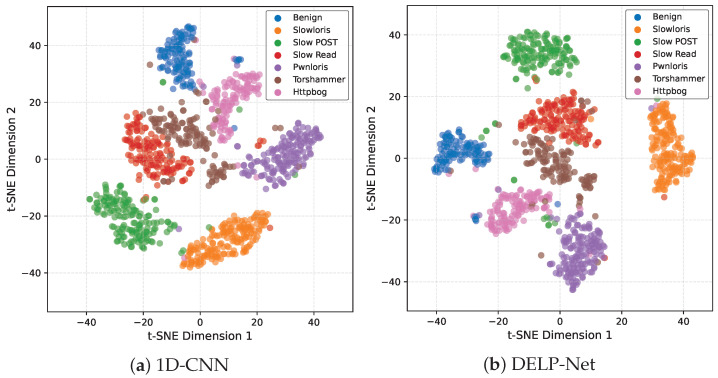
t-SNE visualization of classifier input embeddings for different models.

Although the detection task can be formulated as binary classification, we visualize the learned embeddings under a multi-class setting to examine whether the model can distinguish fine-grained LDoS variants with highly similar low-rate and bursty behaviors. We employ t-distributed stochastic neighbor embedding (t-SNE) to project the high-dimensional classifier input features into a two-dimensional space [60]. Figure 6 compares the resulting feature distributions of the 1D-CNN baseline and the proposed DELP-Net.

In the 1D-CNN embedding, benign windows and multiple LDoS subclasses exhibit substantial overlap, and closely related attacks are poorly separated, indicating that raw-byte convolution is strongly affected by background burstiness and struggles to capture subtle distributional regularities. In contrast, DELP-Net produces more compact intra-class clusters and clearer margins between benign traffic and all attack subclasses while significantly reducing overlap among different low-rate DoS variants. This suggests that DREAM and entropy-regularized training promote an entropy-aware representation space that suppresses high-entropy background noise and emphasizes repetitive low-entropy patterns, resulting in improved separability and more stable decision boundaries.

### 5.6. Computational Cost and Deployment Feasibility

To further evaluate the practicality of DELP-Net for real-time intrusion detection, we analyze its computational cost and deployment efficiency. In particular, we report the number of trainable parameters, approximate computational complexity (FLOPs), inference latency per traffic sample, and memory usage during inference.

All experiments are conducted using the same implementation environment described in Section 4.5. During latency measurement, the model is executed in inference mode with gradient computation disabled. The reported latency corresponds to the average runtime per sample after a warm-up stage.

Table 8 reports the overall model complexity. DELP-Net maintains a moderate number of parameters while avoiding the higher computational overhead typically introduced by deeper sequence models. The additional computation mainly originates from the entropy-aware feature recalibration and temporal modeling modules, which significantly improve detection performance while keeping the overall complexity manageable.

As shown in Table 9, DELP-Net requires only a small inference delay for each traffic sample and is able to process 610 samples per second. Considering that each detection sample corresponds to one flow of traffic in our dataset construction, the measured inference latency is significantly lower than the data acquisition interval. This indicates that DELP-Net can operate in real time without introducing processing bottlenecks.

In addition, the memory consumption remains moderate, which supports the practical deployment of DELP-Net in online IDS environments on conventional GPU-enabled monitoring servers.

### 5.7. Detection Granularity

The proposed method evaluates detection performance at the traffic-sample level, where each sample corresponds to a fixed-length traffic window extracted from network flows. This window-based representation is commonly adopted in intrusion detection studies because it allows continuous monitoring of network behavior while maintaining stable input dimensions for deep learning models.

Although the reported metrics are computed at the sample level, the detection results can be naturally aggregated at the flow or attack-event level in practical deployment. In an online IDS environment, multiple consecutive windows belonging to the same flow or service session are analyzed sequentially. Once a certain number of windows are classified as malicious, the corresponding flow or attack event can be identified. Therefore, the window-level detection used in this work provides a fine-grained and early indication of potential attacks.

In addition, the inference latency analysis reported in Section 5.6 shows that the proposed model can process traffic samples in real time. Since each sample corresponds to a short observation interval, the detection delay for an attack event is limited to only a few windows in practice. This property allows the system to respond to low-rate DoS behaviors before the attack traffic accumulates to a disruptive level.

Regarding traffic load variations, the datasets used in our experiments contain diverse background traffic patterns and protocol distributions, including both public benchmark datasets and traffic collected from a self-built experimental environment with injected jitter and service fluctuations. These characteristics provide a certain degree of robustness evaluation under varying traffic conditions.

## 6. Conclusions

Low-rate Denial-of-Service (LDoS) attacks induce congestion or resource exhaustion at critical moments through periodic traffic pulses. Due to their low average traffic rate and the similarity of their behavior to legitimate bursty traffic, traditional detection methods based on amplitude thresholds or simple statistical features find it difficult to achieve both high detection rates and low false-positive rates under complex background traffic. To address this challenge, this paper proposes an end-to-end differentiable entropy pyramid network, DELP-Net, for window-level low-rate DoS detection directly from raw network traffic, from a joint information-theoretic and deep representation learning perspective. The core idea of the proposed method is to transform information entropy from an external statistical feature into an internal learnable operator and to embed it deeply into feature representation and temporal modeling. Specifically, a differentiable Rényi entropy attention module, DREAM, is designed to explicitly model distributional concentration and repetitive structures in the feature space, enabling the model to effectively perceive entropy anomalies caused by LDoS attacks under low-amplitude conditions. Meanwhile, a multi-scale entropy pyramid learning framework is constructed to jointly model convolutional features and entropy statistics across different receptive-field scales, covering the multi-scale characteristics of LDoS attacks in both local burst behaviors and long-period structures. On this basis, an entropy-conditioned temporal convolutional network is introduced for cross-window temporal modeling, guiding the model to focus on time segments corresponding to entropy anomalies or abrupt entropy variations, so as to stably capture the periodic pulse dependencies of LDoS attacks. In addition, an entropy-regularized hybrid loss function is introduced to further enhance the separability of attack and benign samples in the entropy domain, improving robustness and false-positive control under complex background traffic. Experimental results on public datasets show that DELP-Net achieves stable and significant performance advantages across six LDoS attack types, with an average F1 score of 0.9877 while maintaining a low false-positive rate. Ablation studies verify the effectiveness of DREAM, multi-scale entropy pyramid fusion, and entropy-conditioned temporal modeling in improving overall performance. Moreover, the model mainly relies on convolutional operators, providing good computational efficiency and online inference feasibility, and meeting the basic requirements of real-world network environments for real-time performance and stability.

Future work will focus on two main directions. First, the robustness of DELP-Net against adversarial LDoS attacks will be systematically investigated, with particular attention to adversarially crafted traffic patterns, dynamic attack parameter variations, and evasion strategies. In this context, online adaptive and continual learning mechanisms will also be explored to enhance model robustness while mitigating the risk of catastrophic forgetting. Second, more efficient deployment optimization strategies will be studied to further reduce inference latency and improve real-time processing capability in high-throughput network environments.

## Figures and Tables

**Table 1:** Statistics of the datasets used in the experiments.

Dataset	Total Samples	Benign	Attack	Protocols	Train/Test
CICIDS2017	62,000	31,500	30,500	TCP/UDP	70/30
CICIDS2018	71,000	36,200	34,800	TCP/UDP	70/30
Self-built	28,500	14,800	13,700	TCP	70/30

**Table 2:** Window-Based Preprocessing Configuration.

Item	Value
Window length $T_{w}$	1.0 s
Window stride $T_{s}$	0.5 s
Max packets per window *K*	16
Bytes per packet *L*	256
Sequence length for TCN *M*	16
Minimum packets per window $K_{\min}$	8
Label overlap threshold τ	0.2

**Table 3:** Key Hyperparameters for DELP-Net.

Item	Value
Optimizer	Adam
Initial learning rate	$1\times 10^{-3}$
Batch size	64
Dropout rate	0.3
Rényi parameter α	2
Entropy regularization weight λ	0.05
TCN layers $L_{t}$	4
TCN kernel size $k_{t}$	3
TCN dilation rates	{1,2,4,8}
Early stopping patience	10

**Table 4:** Per-Attack Detection Results of DELP-Net.

Type	ACC (%)	DR (%)	FPR (%)	Precision (%)	F1
Slowloris	98.72	98.96	1.52	98.49	0.9872
Slow POST	98.76	98.60	1.08	98.92	0.9876
Slow Read	98.82	98.56	0.92	99.08	0.9882
Pwnloris	98.54	98.40	1.32	98.68	0.9854
Torshammer	98.90	98.88	1.08	98.92	0.9890
Httpbog	98.88	98.76	1.00	99.00	0.9888
(Average)	98.77	98.69	1.15	98.85	0.9877

**Table 5:** Overall comparison with baselines under window-based evaluation.

Method	ACC (%)	DR (%)	FPR (%)	Precision (%)	F1
Entropy-Threshold	92.63	92.76	7.50	92.52	0.9264
RF	91.98	91.95	7.99	92.01	0.9198
XGBoost	91.64	91.57	8.29	91.70	0.9164
1D-CNN	94.70	94.66	5.25	94.74	0.9470
LSTM	94.83	94.92	5.26	94.75	0.9483
DELP-Net	98.77	98.69	1.15	98.85	0.9877

**Table 6:** Comparison with recent state-of-the-art methods.

Method (Year)	ACC (%)	DR (%)	Precision (%)	F1
FFCNN (2022) [51]	–	96.30	97.50	0.9690
CNN-BMECapSA-RF (2023) [52]	95.91	94.69	94.95	0.9482
GASF-IPP (2023) [53]	93.57	93.22	95.39	0.9429
MSCBL-AND (2024) [54]	96.74	96.74	96.77	0.9675
ERT-EDR (2024) [55]	95.25	97.91	–	0.9557
HawkEye (2025) [56]	91.78	–	96.46	0.9307
DELP-Net (Proposed)	98.77	98.69	98.85	0.9877

**Table 7:** Ablation study results.

Variant	ACC (%)	DR (%)	FPR (%)	Precision (%)	F1
w/o DREAM	94.40	94.28	5.48	94.51	0.9439
DREAM to SE	95.85	95.83	4.13	95.87	0.9585
DREAM to CBAM	96.56	96.61	3.48	96.52	0.9656
w/o Entropy Pyramid Fusion	96.70	96.66	3.26	96.74	0.9670
w/o TCN	97.60	97.87	2.66	97.35	0.9761
TCN w/o Entropy Conditioning	98.20	98.12	1.71	98.28	0.9820
DELP-Net (Full)	98.77	98.69	1.15	98.85	0.9877

**Table 8:** Model complexity comparison.

Model	Parameters (M)	FLOPs (G)
1D-CNN	0.94	0.41
LSTM	1.57	0.68
DELP-Net	1.16	0.47

**Table 9:** Inference efficiency and resource usage.

Model	Latency/Sample (ms)	Samples/s	GPU Memory (MB)
1D-CNN	1.21	826	486
LSTM	2.87	348	664
DELP-Net	1.64	610	528

## Data Availability

The CICIDS2017 dataset used in this research can be retrieved via the following URL: https://www.unb.ca/cic/datasets/ids-2017.html (accessed on 6 February 2026). The CICIDS2018 dataset used in this research can be retrieved via the following URL: https://www.unb.ca/cic/datasets/ids-2018.html (accessed on 6 February 2026).
